# Subtype Identification of Surgically Curable Primary Aldosteronism During Treatment With Mineralocorticoid Receptor Blockade

**DOI:** 10.1161/HYPERTENSIONAHA.124.22721

**Published:** 2024-03-25

**Authors:** Giovanni Pintus, Teresa Maria Seccia, Laurence Amar, Michel Azizi, Anna Riester, Martin Reincke, Jiří Widimský, Mitsuhide Naruse, Tomaz Kocjan, Aurelio Negro, Gregory Kline, Akiyo Tanabe, Fumitoshi Satoh, Lars Christian Rump, Oliver Vonend, Peter J. Fuller, Jun Yang, Nicholas Yong Nian Chee, Steven B. Magill, Zulfiya Shafigullina, Marcus Quinkler, Anna Oliveras, Bo-Ching Lee, Chin-Chen Chang, Vin-Cent Wu, Zuzana Krátká, Michele Battistel, Domenico Bagordo, Brasilina Caroccia, Giulio Ceolotto, Giacomo Rossitto, Gian Paolo Rossi

**Affiliations:** 1 Internal Emergency Medicine Unit, Department of Medicine, Specialized Center for Blood Pressure Disorders-Regione Veneto (G.P., T.M.S., D.B., B.C., G.C., G.R., G.P.R.), University of Padova, Italy.; 2 Institute of Radiology (M.B.), University of Padova, Italy.; 3 Department of Translational Medicine, Sapienza University of Rome, Italy (G.P.).; 4 Université Paris Cité, Institut national de la santé et de la recherche médicale (INSERM) UMRS 970 and CIC1418, France (L.A., M.A.).; 5 Assistance Publique-Hopitaux De Paris Hôpital Européen Georges Pompidou, Hypertension Unit, Paris, France (L.A., M.A.).; 6 Department of Medicine IV, Ludwig Maximilian University of Munich (LMU) University Hospital, LMU Munich (A.R., M.R.).; 7 3^rd^ Department of Medicine (J.W., Z.K.), 1^st^ Faculty of Medicine and General University Hospital, Prague, Czech Republic.; 8 Department of Endocrinology and Metabolism (J.W., Z.K.), 1^st^ Faculty of Medicine and General University Hospital, Prague, Czech Republic.; 9 Department of Endocrinology, Clinical Research Institute, National Hospital Organization Kyoto Medical Center and Endocrine Center, Ijinkai Takeda General Hospital, Japan (M.N.).; 10 University Medical Centre Ljubljana, Faculty of Medicine, University of Ljubljana, Slovenia (T.K.).; 11 Internal Medicine and Hypertension Center, Ospedale Sant’Anna di Castelnovo Ne’ Monti (A.N.).; 12 Azienda Unità sanitaria locale - Istituti di Ricovero e Cura a Carattere Scientifico - (ULS-IRCCS) di Reggio Emilia, Italy (A.N.).; 13 University of Calgary, Foothills Medical Centre, Canada (G.K.).; 14 Department of Diabetes, Endocrinology and Metabolism, National Center for Global Health and Medicine, Tokyo, Japan (A.T.).; 15 Department of Nephrology, Endocrinology and Vascular Medicine, Tohoku University Hospital, Sendai (F.S.).; 16 Department of Nephrology, Medical Faculty, University Hospital Düsseldorf, Heinrich-Heine-University Düsseldorf, Germany (L.C.R., O.V.).; 17 Monash Health, Clayton, VIC, Australia (P.J.F., J.Y., N.Y.N.C.).; 18 Medical College of Wisconsin, Endocrinology Center, North Hills Health Center, Menomonee Falls, WI (S.B.M.).; 19 Department of Endocrinology, North-Western State Medical University named after I.I. Mechnikov, St. Petersburg, Russia (Z.S.).; 20 Endocrinology in Charlottenburg, Berlin, Germany (M.Q.).; 21 Hypertension Unit, Nephrology Department, Hospital del Mar, Universitat Pompeu Fabra, Barcelona, Spain (A.O.).; 22 Department of Medical Imaging, National Taiwan University Hospital, Taipei (B.-C.L., C.-C.C.).; 23 National Taiwan University College of Medicine, Taipei (C.-C.C.).; 24 Department of Internal Medicine, National Taiwan University Hospital and National Taiwan University College of Medicine, Taipei, Taiwan (V.-C.W.).

**Keywords:** aldosterone, blood pressure, hyperaldosteronism, hypertension, mineralocorticoid receptor antagonists, renin

## Abstract

**BACKGROUND:**

Current guidelines and consensus documents recommend withdrawal of mineralocorticoid receptor antagonists (MRAs) before primary aldosteronism (PA) subtyping by adrenal vein sampling (AVS), but this practice can cause severe hypokalemia and uncontrolled high blood pressure. Our aim was to investigate if unilateral PA can be identified by AVS during MRA treatment.

**METHODS:**

We compared the rate of unilateral PA identification between patients with and without MRA treatment in large data sets of patients submitted to AVS while off renin-angiotensin system blockers and β-blockers. In sensitivity analyses, the between-group differences of lateralization index values after propensity score matching and the rate of unilateral PA identification in subgroups with undetectable (≤2 mUI/L), suppressed (<8.2 mUI/L), and unsuppressed (≥8.2 mUI/L) direct renin concentration levels were also evaluated.

**RESULTS:**

Plasma aldosterone concentration, direct renin concentration, and blood pressure values were similar in non-MRA-treated (n=779) and MRA-treated (n=61) patients with PA, but the latter required more antihypertensive agents (*P*=0.001) and showed a higher rate of adrenal nodules (82% versus 67%; *P*=0.022) and adrenalectomy (72% versus 54%; *P*=0.01). However, they exhibited no significant differences in commonly used AVS indices and the area under the receiving operating characteristic curve of lateralization index, both under unstimulated conditions and postcosyntropin. Several sensitivity analyses confirmed these results in propensity score matching adjusted models and in patients with undetectable, or suppressed or unsuppressed renin levels.

**CONCLUSIONS:**

At doses that controlled blood pressure and potassium levels, MRAs did not preclude the identification of unilateral PA at AVS.

**REGISTRATION:**

URL: https://www.clinicaltrials.gov; Unique identifier: NCT01234220.

**NOVELTY AND RELEVANCE**

**What Is New?**

This study showed the feasibility of performing adrenal vein sampling for the subtyping of primary aldosteronism in patients treated with mineralocorticoid receptor antagonists.

**What Is Relevant?**

Mineralocorticoid receptor antagonists prescribed at doses sufficient to control blood pressure or hypokalemia do not preclude the diagnosis of unilateral primary aldosteronism.

**Clinical/Pathophysiological Implications?**

Mineralocorticoid receptor antagonists, when adequately up-titrated, allow control of hypokalemia and high blood pressure values in the vast majority of patients with primary aldosteronism. The possibility of using them before adrenal vein sampling allows to perform the procedure under safer conditions, without endangering the accuracy of unilateral primary aldosteronism identification.

Available guidelines recommend performing adrenal vein sampling (AVS) for the subtyping of patients with primary aldosteronism (PA) when renin is suppressed or low, because unsuppressed renin levels, by stimulating aldosterone secretion in the nonresponsible adrenal gland, would blunt the difference between the responsible and nonresponsible side.^1,2^ Accordingly, an expert consensus statement suggested withdrawal of drugs that stimulate renin, such as diuretics, renin-angiotensin system blockers, and mineralocorticoid receptor antagonists (MRAs), and performing AVS only when plasma renin has returned to suppressed levels.^1,2^ Therefore, it is usually recommended that the MRAs should be withdrawn for 4 to 6 weeks before planning AVS;^1,2^ however, this can put at risk PA patients with stage III hypertension, resistant hypertension particularly if coexisting with severe hypokalemia.

Therefore, it would be much desirable for such patients to be able to undergo AVS during MRA treatment under well-controlled serum potassium and blood pressure (BP) levels. However, the feasibility of this approach remains to be proven in adequately sized studies. Recently, the results of the prospective EMIRA study (Effects of Mineralocorticoid and AT1 Receptor Antagonism on The Aldosterone-Renin Ratio In Primary Aldosteronism) indicated the possibility of avoiding these risks with MRAs administration, administered at doses that effectively controlled BP values and hypokalemia, without jeopardizing the diagnosis of surgically curable PA.^3^ Moreover, observational studies suggested that MRAs would not preclude the identification of unilateral PA (uPA).^4–7^ Therefore, we set out to investigate the hypothesis that MRA treatment does not preclude an unambiguous diagnosis of uPA by means of AVS in the AVIS-2 (Adrenal Vein Sampling International Study 2), a large database of consecutive patients with PA submitted to AVS in referral centers of 4 continents,^8^ and in patients referred to the Hypertension Center of the University of Padua.

## METHODS

### Data Availability

The data that support the findings of this study are available from the corresponding author upon reasonable request.

### Study Population and Definitions

The detailed protocol, methods, and definitions used in AVIS-2 have been already reported^9,10^ and are summarized in the Supplemental Material. Briefly, we evaluated consecutive patients who underwent AVS from 2000 to 2015 in the AVIS-2 study, a large multicenter international study comprising 19 referral centers in 4 continents,^9^ and consecutive patients investigated at the University of Padua from 2016 to 2023. For the present study, exclusion criteria were treatment with drugs that interfere with the RAAS (renin-angiotensin-aldosterone system) other than MRAs, lack of information on MRA therapy, and unavailable data on AVS indices. The diagnostic accuracy of AVS was examined using, as gold reference, an unambiguous diagnosis of uPA following the Standards for Reporting Diagnostic accuracy (STARD) guidelines.^11^ The diagnostic criteria entailed biochemical evidence of PA correction, that is normalization of both hypokalemia without K^+^ supplementation and the aldosterone-to-renin ratio, along with correction of renin suppression (see below) after removal of the responsible adrenal gland.^9^ The patients who showed no lateralization at AVS or no biochemical cure after surgery, were presumed to have non-uPA.^12^

### Study Aims and End Points

The primary aim was to compare the rate of uPA identification by the lateralization index (LI) between patients treated or untreated with MRAs by AVS. Secondary aims were (1) to evaluate the effect of MRAs on 2 AVS indices, the LI and the relative aldosterone secretion index and (2) to investigate the accuracy of subtyping PA with AVS as a function of the level of renin suppression.

When the primary end point was scrutinized, only bilaterally selective procedures were selected, for both unstimulated and postcosyntropin stimulation procedures. For the secondary aims, LI required bilaterally selective procedures, while selectivity only on the responsible or the contralateral side was needed when examining the relative aldosterone secretion index (Table S1). As an overall index of accuracy, we used the area under the receiver operating characteristic (ROC) curve (AUROC) using uPA as categorical status.

To overcome the limitations intrinsic to the retrospective nature of the study and the lack of randomization, we performed propensity score matching (PSM), as described in detail in the Supplemental Material.^13^ To rule out differences in the LI and the rate of lateralization at AVS between the MRA and non-MRA group due to confounders, we built 4 different PSM models according to the type of value sampling and tolerance, number of covariates studied or the handling of missing values.

Moreover, to test the hypothesis that unsuppressed plasma renin levels could interfere with uPA identification and ascertainment of lateralization, we performed a sensitivity analysis comparing the AUROCs for uPA identification and LI in patients with undetectable (DRC, ≤2 mUI/L),^2^ suppressed (<8.2 mUI/L) and unsuppressed (≥8.2 mUI/L) renin levels.^11,12^

## RESULTS

### Baseline Characteristics of the Population

**Figure 1.**
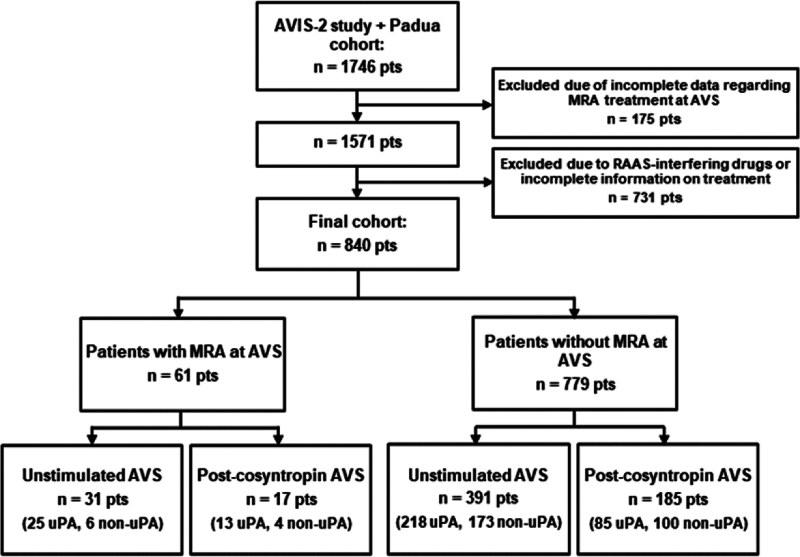
**Flowchart of the study.** The flowchart depicts the selection of the patients for this study. AVS indicates adrenal vein sampling; AVIS-2, Adrenal Vein Sampling International Study 2; MRA, mineralocorticoid receptor antagonist; RAAS renin-angiotensin-aldosterone system; and uPA, unilateral primary aldosteronism.

We recruited for this study 1746 consecutive patients with PA submitted to AVS, 1625 were from the original AVIS-2 study cohort, and 121 were consecutive PA patients in Padua after the cohort originally included in the AVIS-2 study (Table S2). After the exclusion of 906 patients because of incomplete data or inadequate pharmacological washout from drugs interfering with the RAAS, we were left with 61 patients submitted to AVS (unstimulated and/or postcosyntropin stimulation) while on MRAs (31 from AVIS-2 and 30 from Padua), and 779 patients (699 from AVIS-2 and 80 from Padua) off MRAs (Figure 1).

Table 1 shows that there were no significant differences of plasma aldosterone concentration, direct renin concentration (DRC), aldosterone renin/ratio, systolic, and diastolic BP values between the MRA and non-MRA groups at the time of AVS. Overall, the serum potassium levels showed only a trend toward higher values in the MRA-treated patients, but a subanalysis of the Padua cohort evidenced higher levels in the MRA- than in the non-MRA-treated patients (3.7 versus 3.4 mEq/L; *P*=0.028). Table 1 also shows that the MRA-treated patients required a more intense antihypertensive treatment (defined daily dose 2.25 versus 1.5; *P*=0.001) and had more adrenal nodules (82 versus 67%; *P*=0.024) at imaging, notwithstanding a similar dimension of the identified nodules (16.3 versus 15.6 mm; *P*=0.642).

**Table 1.**
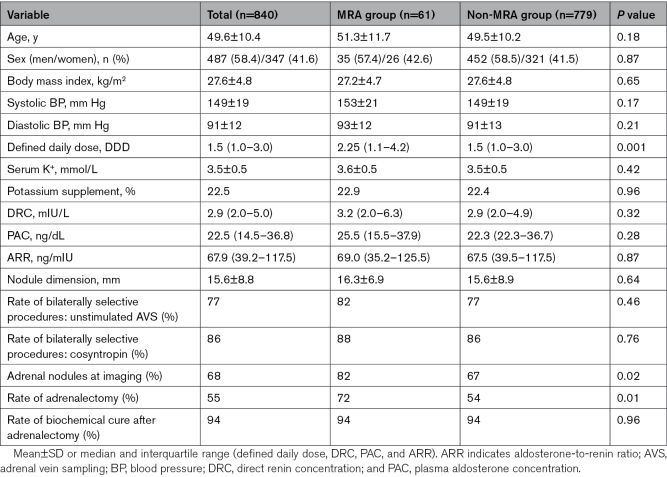
Baseline Demographic, Clinical, and Biochemical Features of All the 840 Patients With PA Included in the Study, Subsequently Divided According to the Presence of MRAs at AVS

### Final Diagnosis and Outcomes

The rate of assignment to medical or surgical treatment differed between the groups: the rate of adrenalectomy was higher in the MRA than the non-MRA group (72 versus 54%, χ^2^=6.588; *P*=0.010). Complete postsurgery follow-up biochemical data, which were available in 64% of all adrenalectomized patients, showed an identical rate of biochemical cure (94%) in the 2 groups.

### Rate of Bilaterally Selective AVS

The rate of bilaterally selective procedures was identical in the two groups under unstimulated conditions (75%), and similar (84 versus 86%; *P*=0.761) postcosyntropin stimulation. The rate of selective procedures was higher on the left than the right side in the whole cohort (unstimulated 87% versus 83%, *P*<0.012; postcosyntropin stimulation 99% versus 87%, *P*<0.001); no differences in the rate of selectivity were found when comparing each side between the MRA and non-MRA groups (Table S3).

### Comparison of Receiver Operator Characteristic Curves

**Figure 2.**
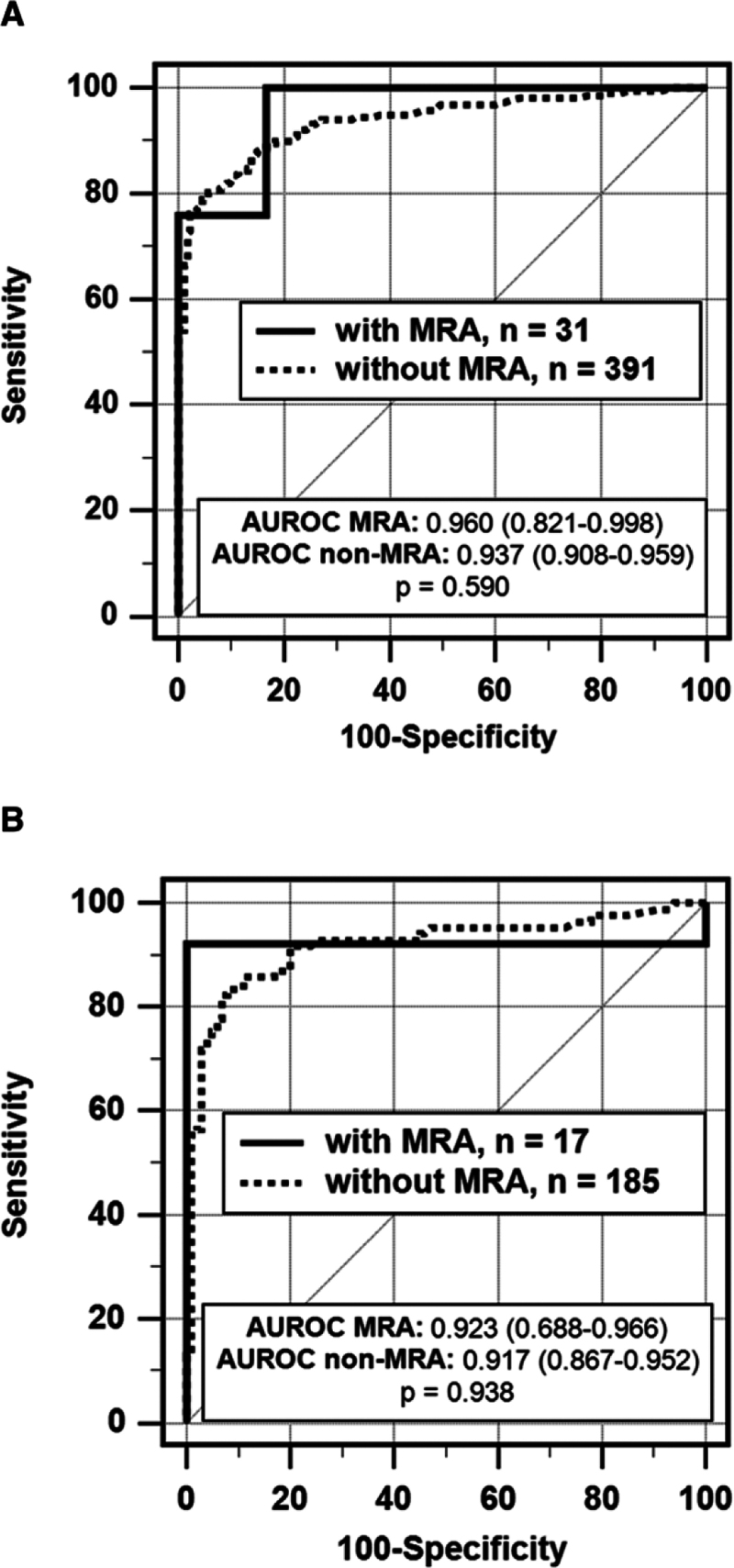
**Receiving operating characteristic (ROC) curves by MRA treatment.** Comparison of ROC curves for lateralization index (LI) under unstimulated conditions (**A**) and postcosyntropin stimulation (B) with vs without MRA treatment. The accuracy of the LI for diagnosing unilateral PA was evaluated by area under ROC curve (AUROC) values (inset). *P* value refers to AUROCs comparison between the MRA and the non-MRA groups. **A**, Unstimulated adrenal vein sampling (AVS) accuracy. **B**, Postcosyntropin AVS accuracy.

The overall accuracy (AUROC) of AVS for the identification of uPA showed no differences (*P*=0.590) between the 31 patients undergoing unstimulated AVS while on MRA therapy (25 uPA and 6 non-uPA; 0.960 [95% CI, 0.821–0.998]) and the 391 patients (218 uPA and 173 non-uPA) without MRA (0.937 [95% CI, 0.908–0.959]; Figure 2; Table 3). The postcosyntropin AUROCs results were also similar between the MRA group (n=17, 13 uPA and 4 non-uPA) and non-MRA group (n=185, 85 uPA and 100 non-uPA) (MRA group: 0.923 [95% CI, 0.688–0.966]; non-MRA group: 0.917 [95% CI, 0.867–0.952]; *P*=0.938).

**Table 2.**
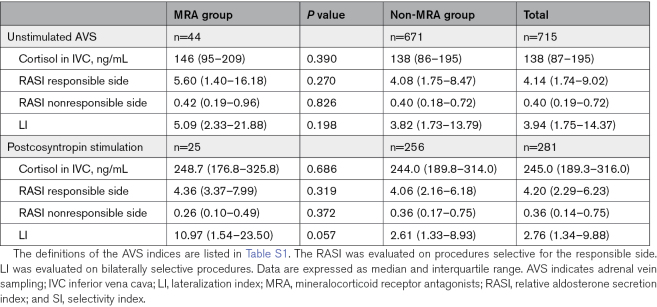
AVS Indices in the MRA and Non-MRA Groups

**Table 3.**
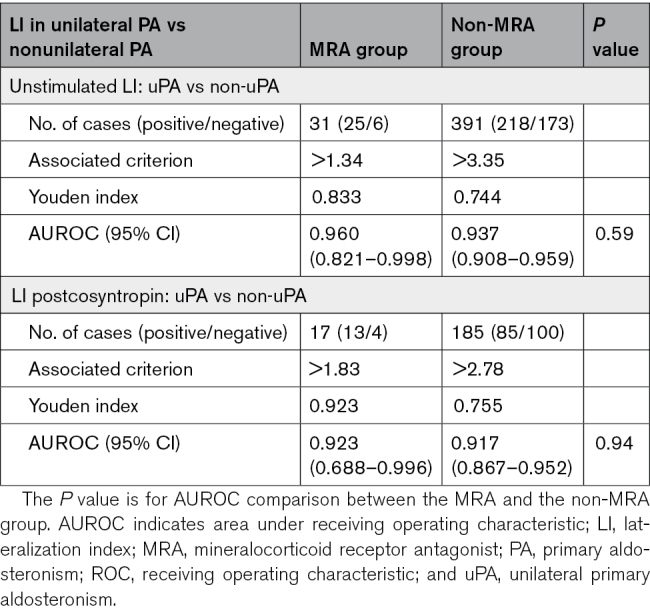
ROC Analyses in Patients With PA for Identification of uPA Using the LI Under Unstimulated Conditions and Postcosyntropin Stimulation, in the MRA and Non-MRA Groups

In line with these results, all the AVS indices showed no significant differences between groups, both with unstimulated conditions and after cosyntropin stimulation (Table 2).

### Propensity Score Matching

After PSM, we found no significant differences in any of the putative variables confounders. We built 4 different models for the PSM (Figure S1). In all models, the LI showed no significant differences between the MRA and non-MRA groups (Supplemental Material). In model 1, the rate of lateralization at AVS was slightly higher in the MRA group (χ^2^=4.7; *P*=0.03), but this was not confirmed in models 2 and 3 (Supplemental Material). The conditional regression analysis in models 1, 2, and 3 showed only a trend toward a higher lateralization rate in the MRA group (Supplemental Material). No significant differences in LI and lateralization rate between the MRA and non-MRA groups was seen in model 4.

### Effect of Renin Suppression on AUROC

**Figure 3.**
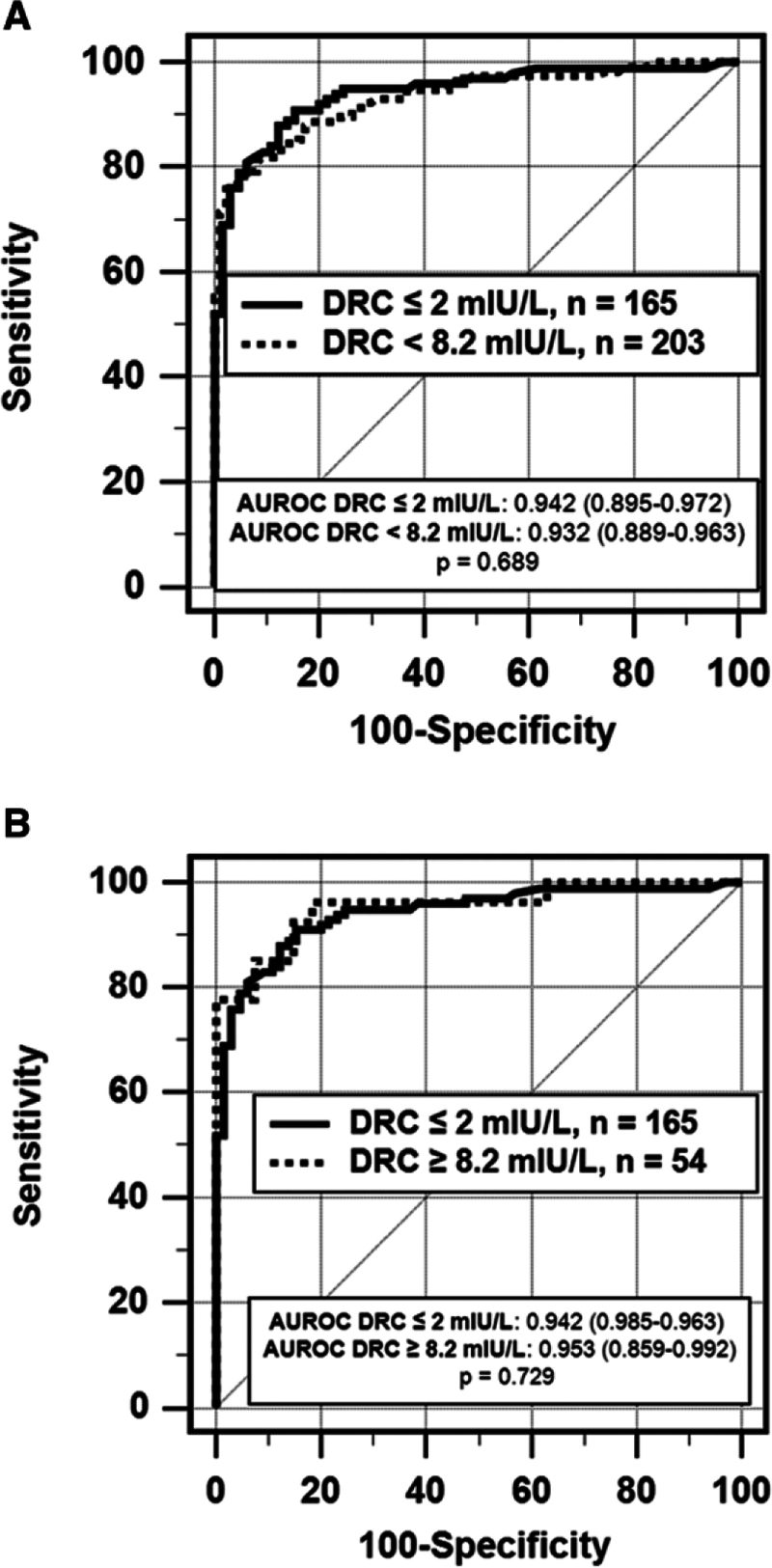
**Receiving operating characteristic (ROC) curves by renin levels.** Diagnostic accuracy (AUROC [area under ROC curve] values) of lateralization index (LI) in identifying unequivocally unilateral PA, vs nonunilateral PA as evaluated with ROC curve. *P* value referees to AUROC comparison between the suppressed renin levels (**A**) and unsuppressed renin levels (**B**) groups. **A**, Unstimulated adrenal vein sampling (AVS) accuracy: undetectable vs suppressed renin levels. **B**, Unstimulated AVS accuracy: undetectable vs unsuppressed renin levels.

Different sensitivity analyses were undertaken to verify if the plasma level of active renin at the time of AVS affected the accuracy of the procedure. The patients were split into groups according to the presence of undetectable (≤2 mUI/L, n=165), suppressed (<8.2 mUI/L, n=203), or unsuppressed (≥8.2 mUI/L n=54) DRC. ROC curves for the LI values were built for each DRC subgroup, using uPA as categorical status. The comparison of the renin undetectable group versus renin suppressed and unsuppressed groups showed no significant differences of AUROCs (*P*=0.689 and 0.729, respectively), thus, demonstrating lack of differences in uPA identification between patients with different DRC levels (Figure 3A and 3B; Table S4). Likewise, there were no significant differences in the LI (Table S5) and the rate of uPA identification between MRA and non-MRA patients in the suppressed and unsuppressed DRC subgroups (Supplemental Material).

## DISCUSSION

PA can present with difficult to control hypokalemia and span from mild (stage I) arterial hypertension, to moderate (stage II), severe (stage III), or drug-resistant hypertension^14^ with prominent organ damage.^15^ Although temporary withdrawal of the antihypertensive therapy or switching to noninterfering drugs were reported to be safe in patients with hypertension without PA,^16^ this is unlikely to apply to those with PA and stage III, resistant hypertension or severe hypokalemia, where MRA treatment is key for controlling these clinical phenotypes and minimizing their risks.

On the other hand, when pursuing surgical treatment, which can be lifesaving in that it resolves drug-resistant hypertension and regresses hypertension-mediated organ damage, unambiguous identification of uPA by AVS is fundamental.^17,18^ Hence, current guidelines recommend withdrawal of MRA for 4 to 6 weeks in patients with a florid clinical phenotype of PA when planning subtyping of PA by AVS,^1,2^ an undertaking that can expose patients with PA to risks of uncontrolled BP, hypokalemia, and thus to cardiovascular events.

### Biochemical and Clinical Features of MRA and Non-MRA-Treated Patients

We first examined if in this study the MRA- and the non-MRA-treated patients with PA differed biochemically and clinically. This comparison showed that the plasma levels of aldosterone, active renin, the aldosterone-renin ratio and the BP values did not differ between the groups; to some extent unexpectedly, serum potassium levels were also similar in the 2 groups (Table 1). However, in the Padua cohort, serum potassium levels were higher (*P*=0.028) in the MRA-treated than in the non-MRA-treated patients, probably because the MRA were systematically up-titrated to achieve normokalemia.^19^ On the whole, the MRA-treated group required more antihypertensive drugs, besides the MRA, to achieve better BP control, and showed a higher rate of nodules at imaging. Thus, they had a more severe PA phenotype in line with the findings of a small study^7^ and of the JPAS study.^6^ These findings can suggest the patients with uPA requiring MRA treatment, who usually show a more florid phenotype, could be more easily identified at AVS than the non-MRA patients.

### Identification of uPA in MRA and Non-MRA Patients

Of considerable importance, our results showed no differences in the rate of identification of uPA between patients on MRA and patients who had withdrawn these drugs at the time of AVS. Moreover, the values of the LI and the relative aldosterone secretion index in the responsible and the nonresponsible side were remarkably similar (Table 2) in the 2 groups, thus, reinforcing the conclusion that MRA treatment does not impact negatively on the assessment of lateralization of aldosterone secretion and, therefore, on the clinical decision making. We consider this demonstration a major advancement in the diagnostic work-up and management of PA.

Evidence obtained in smaller studies also supports the conclusion that MRA treatment did not endanger the identification of uPA: in 2014, Haase et al^5^ described 4 patients with florid PA and suppressed renin levels during MRA treatment, who showed lateralization at AVS, and were eventually cured with adrenalectomy. In 2019, Nanba et al^4^ reported no significant differences in AVS indices and complete biochemical success after adrenalectomy in 22/23 MRA-treated patients. Similarly, in 2020, Ganesh et al^7^ reported no significant differences in biochemical outcomes between 5 patients who underwent AVS during MRA treatment and 12 patients without MRA. Torresan et al^17^ more recently showed the feasibility of ascertaining uPA by means of AVS in patients with resistant hypertension, who, by definition, were on multiple RAAS-interfering drugs. Of note, in that study, 75% of the patients were on MRA treatment and had a median DRC of 2 mUI/L. Thus, in line with previous reports, the results of this study support the contention that MRAs should not be withdrawn before AVS, even if plasma renin is not suppressed.^4–6^

### Analysis by Plasma Renin Levels

In the previous and current guidelines, recommendation of MRA withdrawal stands on the theoretical possibility that the unsuppressed renin levels would blunt the LI by enhancing aldosterone secretion in the nonresponsible adrenal.^1^ Accordingly, in 2014, a panel of experts stated that patients with unsuppressed renin levels^20^ should not be submitted to AVS. This was because AVS is an expensive and (minimally) invasive procedure, which, therefore, should be performed only under optimal conditions. However, this recommendation was either not graded,^1,20^ or based on a low level of evidence (class of recommendation II and level of evidence B),^2^ and is now questioned by studies published thereafter^3–6^ as discussed below.

To challenge this recommendation, we performed sensitivity analyses looking at the identification of uPA in patients submitted to AVS while having undetectable, suppressed, or unsuppressed renin levels. These analyses showed no significant differences of uPA identification and the LI-based AUROCs for uPA in any of these subgroups (Table S4), indicating that complete renin suppression is not a conditio sine qua non for identification of uPA when planning AVS in patients with PA. Some caution is advised in generalizing this conclusion to the patients with PA with unsuppressed renin, that is, DRC ≥8.2 mIU/L, because there were only 5 in the MRA group. A recent prospective study showed that high DRC values are unlikely in patients with proven PA: after 1 month of MRA treatment up-titrated to control high BP and hypokalemia, no such patients had DRC values >9 mIU/L in the surgically cured group and only 2 had values between 8 and 11 mIU/L in the (less florid) medically treated patients.^3^

From the practical standpoint, these findings have 2 important implications: (1) they rule out the need to remeasure DRC before AVS in patients with PA judged to be eligible for the procedure and (2) they suggest that withdrawal of MRAs to bring renin down to suppressed levels before AVS is probably unnecessary. These conclusions might not apply to the patients on ACE (angiotensin-converting enzyme) inhibitors and ARBs (angiotensin type 1 receptor blockers), who were excluded from the present study. However, in the JPAS study of patients with PA on multiple drugs, 58% of whom were on an ACE inhibitors or ARB, a cosyntropin-stimulated LI >4 predicted biochemical cure after adrenalectomy.^6^

### Effect of Cosyntropin Stimulation

Despite impacting negatively on the identification of uPA lateralization at AVS in several cases,^21^ cosyntropin stimulation is still broadly used during AVS.^22^ To investigate whether this stimulation affected AVS performed during MRA treatment, in a sensitivity analysis of 202 patients submitted to cosyntropin-stimulated AVS, we compared the AUROC of MRA- and non-MRA-treated patients. We found that MRA treatment did not compromise the identification of uPA by AVS, in line with what observed in unstimulated AVS.

### Limitations and Strengths

We acknowledge that the retrospective design and the lack of randomization might have led to an unbalanced distribution of potential confounders.^13^ To address this potential limitation, we performed a propensity score matching, which showed no differences in the LI between the MRA and non-MRA group, and even a trend toward a higher rate of lateralization in the MRA group (Supplemental Material).

Another limitation common to all studies on PA regards the diagnosis of nonunilateral PA, which remains only presumptive owing to the lack of surgical confirmation and of gold-standard diagnostic reference for this condition. Theoretically, the ideal study design should involve a prospective study with repeating AVS in the same patients with and without MRAs and submitting all patients with PA to unilateral adrenalectomy, two practices clearly not feasible because they would be considered unethical. A further limitation of this study might be a type II error due to a limited power, because by applying strict exclusion criteria, we were left with 61 patients on MRA. This low rate of AVS on MRA reflects adherence to the current guidelines recommendations of stopping these drugs,^1,2^ in this size real-world study, which depicts current practice in the subtyping of PA. Moreover, this seemingly small number exceeds by 2 to 3-fold that examined in the previous studies^4,5^ of MRAs treatment during AVS. Finally, because of the strict selection of the patients and the ensuing relatively small size, different ethnicities were poorly represented in this study and, therefore, it might be argued that the lack of effect of MRA treatment of AVS results cannot be generalized. However, the results of a study performed in Japanese patients on multiple drugs suggest that the lack of interference by drug treatment apply also to Asiatic patients with PA.^6^

In conclusion, this study provided evidence that MRA treatment, which effectively controls high BP and hypokalemia in most of the patients with PA, did not preclude the identification of uPA and, therefore, should not be withdrawn in patients undergoing AVS.

### PERSPECTIVES

Many patients with PA are currently denied subtyping by AVS and a definitive cure with adrenalectomy due to concerns that stopping MRA treatment could cause severe hypokalemia or uncontrolled BP. The results of this study show the feasibility of diagnosing uPA by AVS without withdrawal of MRAs even if they showed unsuppressed plasma renin levels. Whether the same holds true in patients with PA who need treatment with additional agents that can stimulate renin secretion needs to be investigated in a further study.

## ARTICLE INFORMATION

### Sources of Funding

This study was supported, in part, by research grants to G.P. Rossi, T.M. Seccia, and G. Rossitto from the Department of Medicine Dotazione Ordinaria della Ricerca, University of Padua, and FORICA (the Foundation for Advanced Research in Hypertension and Cardiovascular Diseases). None of the sponsors had any role in the study design, collection, analysis, interpretation of data, in the writing of the report, and in the decision to submit the article for publication.

### Disclosures

None.

## Supplementary Material
