# Supplementary material for: Subtype Identification of Surgically Curable Primary Aldosteronism During Treatment With Mineralocorticoid Receptor Blockade
Source: Hypertension. 2024 Mar 25;81(6):1391–9. doi: 10.1161/HYPERTENSIONAHA.124.22721 (PMC11095898; doi:10.1161/HYPERTENSIONAHA.124.22721)

**Subtype Identification of Surgically Curable Primary Aldosteronism during Treatment with Mineralocorticoid Receptor Blockade**

**Short title:** MRA during Primary Aldosteronism Subtyping

Giovanni Pintus^1,2^ MD; Teresa Maria Seccia MD, PhD^1^, Laurence Amar MD, PhD^3^; Michel Azizi MD, PhD^3^; Anna Riester MD, PhD^4^; Martin Reincke MD^4^; Jiří Widimský MD^5^; Mitsuhide Naruse MD^6^; Tomaz Kocjan MD^7^; Aurelio Negro MD^8^, Gregory Kline MD^9^; Akiyo Tanabe MD, PhD^10^; Fumitoshi Satoh MD^11^; Lars Christian Rump MD^12^; Oliver Vonend MD^12^; Peter J. Fuller MD, PhD^13^; Jun Yang MD^13^; Nicholas Yong Nian Chee MD^13^; Steven B. Magill MD^14^; Zulfiya Shafigullina MD^15^; Marcus Quinkler MD^16^, Anna Oliveras MD^17^; Bo-Ching Lee MD^18^; Chin-Chen Chang MD^18,19^; Vin-Cent Wu MD^20^; Zuzana Krátká MD^5^; Michele Battistel MD^21^; Domenico Bagordo MD^1^; Brasilina Caroccia, PhD^1^; Giulio Ceolotto PhD^1^, Giacomo Rossitto MD, PhD^1^, Gian Paolo Rossi MD^1^;

^1^ University of Padova, Department of Medicine-DIMED, Hypertension Unit, University Hospital, Padova, Italy;

^2^ Sapienza University of Rome, Department of Translational Medicine, Rome, Italy

^3^ Université Paris Cité, INSERM UMRS 970 and CIC1418, F-75015 Paris, France; AP-HP, Hôpital Européen Georges Pompidou, Hypertension Unit, F-75015 Paris, France,

^4^ Department of Medicine IV, LMU University Hospital, LMU Munich

^5^ 3^rd^ Department of Medicine – Department of Endocrinology and Metabolism, 1^st^ Faculty of Medicine and General University Hospital, Prague, Czech Republic

^6^ Dept. of Endocrinology Clinical Research Institute, National Hospital Organization Kyoto Medical Center and Endocrine Center, Ijinkai Takeda General Hospital, Kyoto, Japan;

^7^ University Medical Centre Ljubljana; Ljubljana; Slovenia; Faculty of Medicine, University of Ljubljana, Ljubljana, Slovenia

^8^ Internal Medicine and Hypertension Center, Ospedale Sant'Anna di Castelnovo Ne' Monti; Azienda Usl-IRCCS di Reggio Emilia;

^9^ University of Calgary, Foothills Medical Centre, Calgary, Canada;

^10^ Department of Diabetes, Endocrinology and Metabolism, National Center for Global Health and Medicine, Tokyo, Japan;

^11^ Tohoku University Hospital, Dept. of Nephrology, Endocrinology and Vascular Medicine, Sendai,

^12^ Department of Nephrology, Medical Faculty, University Hospital Düsseldorf, Heinrich-Heine-University Düsseldorf, Germany;

^13^ Monash Health, Clayton, VIC 3168 Australia;

^14^ Medical College of Wisconsin. Endocrinology Center. North Hills Health Center, Menomonee Falls, WI 53051;

^15^ Department of Endocrinology, North-Western State Medical University Named after I.I Mechnikov, St. Petersburg, Russia;

^16^ Endocrinology in Charlottenburg, Berlin, Germany;

^17^ Hypertension Unit, Nephrology Department, Hospital del Mar, Universitat Pompeu Fabra, Barcelona, Spain;

^18^ Department of Medical Imaging, National Taiwan University Hospital, Taipei, Taiwan;

^18,19^ National Taiwan University College of Medicine, Taipei, Taiwan;

^20^ Department of Internal Medicine, National Taiwan University Hospital and National Taiwan University College of Medicine, Taipei, Taiwan;

^21^ University of Padua, Institute of Radiology, Padova, Italy;

Please note that some authors have multiple affiliations.

The AVIS was registered at clinicaltrials.gov number NCT01234220.

**Corresponding Author**

Prof. Gian Paolo Rossi, MD. FACC, FAHA.

Internal Emergency Medicine Unit, Department of Medicine - DIMED University of Padua, Specialized Center for Blood Pressure Disorders-Regione Veneto, 35128 Padua, Italy

Phone: +39-049-821.2279;

E-mail: gianpaolo.rossi@unipd.

**SUPPLEMENTAL MATERIAL**

| **Materials and Methods** | **Pag 4** |
| --- | --- |
| **Distributions of the putative confounders for each model of PSM by MRA treatment** | **Pag 7** |
| **Lateralization index (LI), the rate of lateralization by chi-square test and by conditional regression model, after propensity score matching (PSM).** | **Pag 11** |
| **Rate of uPA identification in MRA and non-MRA patients by DRC subgroup in bilaterally selective and unstimulated AVS: A) undetectable renin levels (DRC ≤ 2 mIU/L); B) suppressed renin levels (DRC < 8.2 mIU/L); C) unsuppressed renin levels (DRC ≥ 8.2 mIU/L).** | **Pag 17** |
| **Supplemental References** | **Pag 20** |
| **Table S1. AVS indexes definitions.** | **Pag 21** |
| **Table S2. Demographic and biochemical features of the PA patients of the Padua and the AVIS-2 cohort.** | **Pag 22** |
| **Table S3. Rate of selective procedures per side** | **Pag 23** |
| **Table S4. Comparison of AUROC for LI under unstimulated conditions by active renin (DRC) levels subgroups: undetectable (≤ 2 mUI/L), suppressed (< 8.2 mUI/L) and unsuppressed (≥ 8.2 mUI/L).** | **Pag 24** |
| **Table S5. Lateralization index (LI) in MRA versus non-MRA patients by active renin (DRC) level subgroups: undetectable (≤ 2 mUI/L), suppressed (< 8.2 mUI/L) and unsuppressed (≥ 8.2 mUI/L).** | **Pag 25** |
| **Table S6. Baseline features of the 121 PA patients from the Padua cohort, subsequently divided according to the presence of MRAs at AVS.** | **Pag 26** |
| **Table S7. Comparison of AUROC for LI under unstimulated conditions by MRA treatment, in PA patients recruited in the Padua cohort.** | **Pag 26** |
| **Figure S1. Propensity score matching flow-chart.** | **Pag 27** |

**MATERIALS AND METHODS**

**Protocol and Study Population**

We retrospectively evaluated 1625 patients of the AVIS-2 study, and 121 patients who underwent AVS between 2016 and 2023 in the University of Padua (Padua 2016-2023 cohort).

1. Complete details for the AVIS2 study were published elsewhere (<https://www.ahajournals.org/doi/full/10.1161/HYPERTENSIONAHA.119.13463>). In brief, the inclusion criteria were a) patients with age ≥18 years; b) agreement to participate in the data collection; c) written informed consent; d) local ethics committee approval. The only exclusion criteria were unwillingness of the lead investigator to participate and/or lack of local ethical/institutional approval.
2. For the Padua cohort, the inclusion criteria were a) PA patients with age ≥18 years; b) agreement to participate in the data collection with written informed consent. PA was diagnosed with a positive ARR test (> 20.06 ng/mUI), if needed repeated one time for confirmation as previously described.

After merging the two databases, patients undergoing treatment with RAAS-interfering drugs, except for MRAs, and patients with insufficient data regarding antihypertensive therapy or AVS indexes were excluded (Figure 1). RAAS-interfering drugs were the following: ACE-inhibitors, angiotensin receptor 2 blockers, beta-blockers, adrenergic agonists, diuretics, renin inhibitors. Drugs permitted were mineralocorticoid receptor antagonists, calcium-channel blockers, alpha-blockers. Defined daily dose (DDD) was defined as reported on WHO website (<https://www.whocc.no/atc_ddd_index/>). For MRAs, 1 DDD corresponded to: Spironolactone 75 mg; Eplerenone 50 mg; Canrenone 50 mg; Potassium Canrenoate 400 mg.

For centers where only Plasma Renin Activity (PRA, in ng/ml/h) values were measured, Direct (Active) Renin Concentration (DRC) was calculated using the ARR-App; the DRC lower limit was set to 2 mIU/L. Plasma aldosterone concentration (PAC) was converted to pmol/L to ng/dl. The aldosterone renin ratio (ARR) was expressed in ng/mUI (from ng/dl/mUI/L * 10).

**Diagnostic accuracy and ROC curve**

For the primary endpoint, the analysis was performed only on bilaterally selective procedures. In brief, a receiver operating characteristic curve (ROC) for the variable LI was built and the area under the ROC curve (AUROC) was calculated. Different ROC curves were built for the unstimulated and post-cosyntropin stimulation. The outcome (classification variable) was defined as it follows: (1) unequivocal diagnosis of unilateral PA, defined as biochemical evidence of correction of the aldosterone excess, according to definition used in the AVIS-2 study, which required at the follow-up visits after adrenalectomy, the normalization of hypokalemia (without supplementation) and aldosterone-to-renin ratio; (2) diagnosis of non-unilateral PA, defined by either absence of biochemical cure after adrenalectomy or absence of lateralization at AVS. The term “non-unilateral PA” was used, considering that the absence of lateralization at AVS does not hold an unequivocal diagnosis of bilateral disease. The study followed the STARD guidelines^1^.

The evaluation of differences in RASI needed the procedures to be selective only on the responsible side.

**AVS protocols and Centers involved in the study**

The AVIS-2 study (Adrenal Vein sampling International Study) is a multi-center registry of patients who underwent AVS, performed in 19 centers worldwide. Further details can be retrieved elsewhere^3-4^. In order to achieve an adequate number of patients to demonstrate our hypothesis, we recruited in the study all the patients that consecutively underwent AVS in our center (University of Padua) starting right after the end of the AVIS study.

Procedures were carried out according to the Helsinki Declaration and the study protocol was approved by the Ethics Committee of the coordinating and participating centers.

**Statistical analysis**

Results are expressed as mean +/- SD, or median and interquartile range, as appropriate. Kolmogorov-Smirnov test was performed to verify normal or skewed distribution, a T-test or Mann-Whitney test were performed to compare normally and non-normally distributed values. Analysis between categorical variables was performed using the chi-square test. Diagnostic performance of AVS variables were analysed with the area under the receiving operator curve (AUROC). Backward (Wald) stepwise regression was used to identify the predictors for AVS lateralization in order not to miss identification of relevant predictors, lateralization index and direct renin concentration were converted in base-10 logarithmic scale. Significance was set at P < 0.05. MedCalc (MedCalc Software, Ostend, Belgium, version 15.8) and SPSS (version 29 for PC; SPSS, Bologna, Italy) were used for data analysis.

**Propensity score matching**

Propensity score matching (PSM) was used to overcome the limitations related to the retrospective nature of the study, and the lack of randomization, which can lead to significant differences between groups at the baseline. We built different models (listed below), according to the type of sampling and/or tolerance (i.e., caliper width, which was derived from the following equation: caliper width = 0.2*standard deviation of the probability of assignment to the MRA or the non-MRA group, the latter calculated by logistic regression), the number of variables studied or the analysis of missing values.

Model 1: PSM with matching 1-to-1, without replacement, no randomized case order, caliper width 0.01; variables were age, sex, serum potassium at AVS, systolic blood pressure.

Model 2: PSM with matching 1-to-1, without replacement, no randomized case order, caliper width 0.02; variables were age, sex, serum potassium at AVS, systolic blood pressure.

Model 3: PSM with matching 1-to-1, with replacement, randomized case order, caliper width 0.01; variables were age, sex, serum potassium at AVS, systolic blood pressure.

Model 4: PSM with matching 1-to-3, with replacement, randomized case order, caliper width 0.01; variables were age, sex, serum potassium at AVS, systolic blood pressure, imaging positive for nodules, defined daily dose; missing values were calculated using the mean of the series.

Subsequently, two groups (MRA versus non-MRA) could be examined, balanced for potential determinants of lateralization at AVS. To evaluate if the two groups were balanced for the chosen variables, we compared their distributions by performing box-and-whisker plots^(2)^. Then, the following statistical analyses were applied: for the lateralization index (LI), a Mann-Whitney test was performed; for the rate of lateralization of AVS procedures, a conditional regression model was performed following using the indication on the official webpage of SPSS (<https://www.ibm.com/support/pages/conditional-logistic-regression-using-coxreg>).

**Distributions of the putative confounders for each model of PSM by MRA treatment**

Model 1. Matching 1:1. Variables were age, sex, systolic blood pressure (SBP), serum potassium.


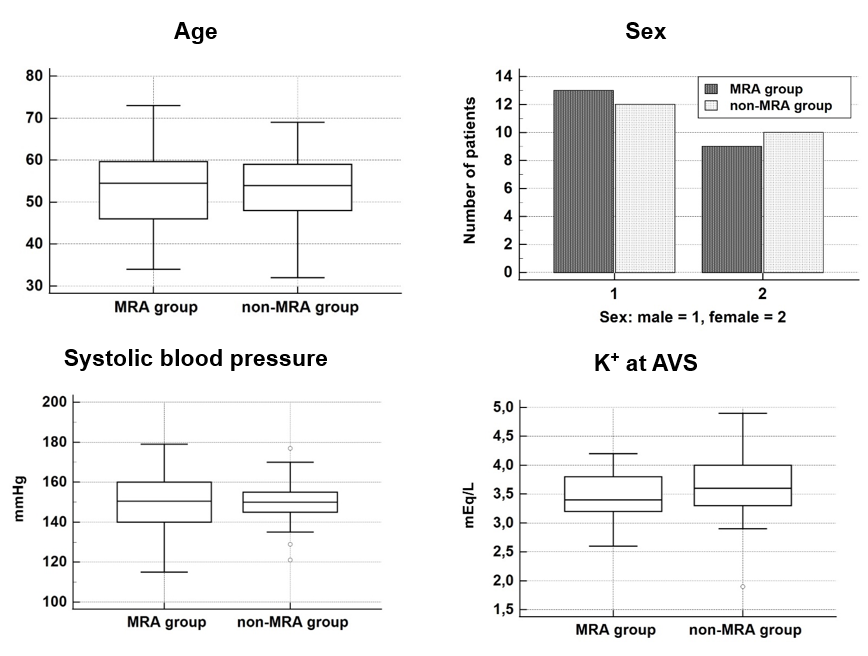


Model 2. Matching 1:1. Variables were age, sex, systolic blood pressure (SBP), serum potassium.


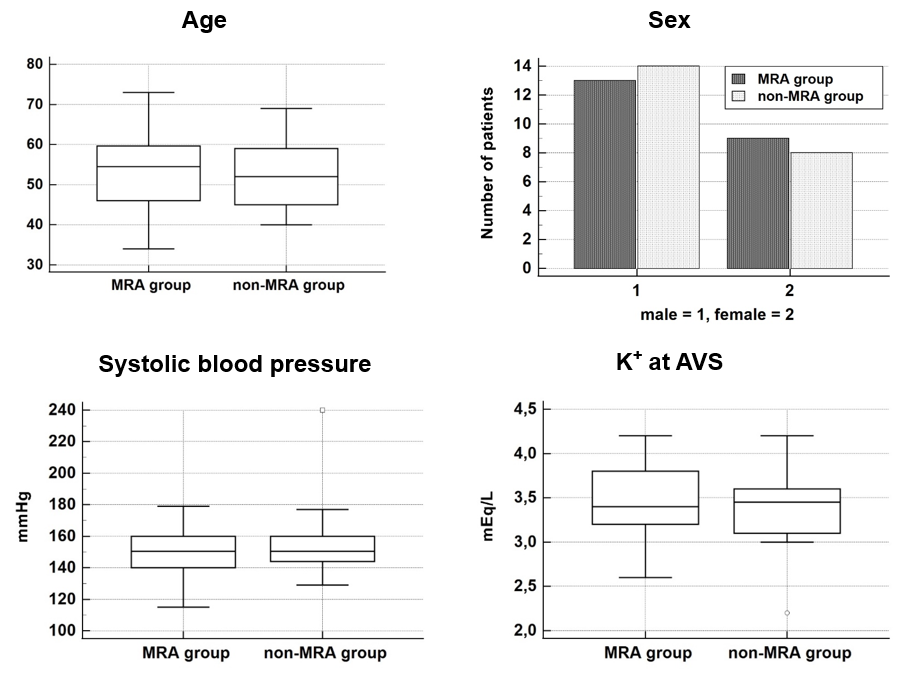


Model 3. Matching 1:1. Variables were age, sex, systolic blood pressure (SBP), serum potassium.


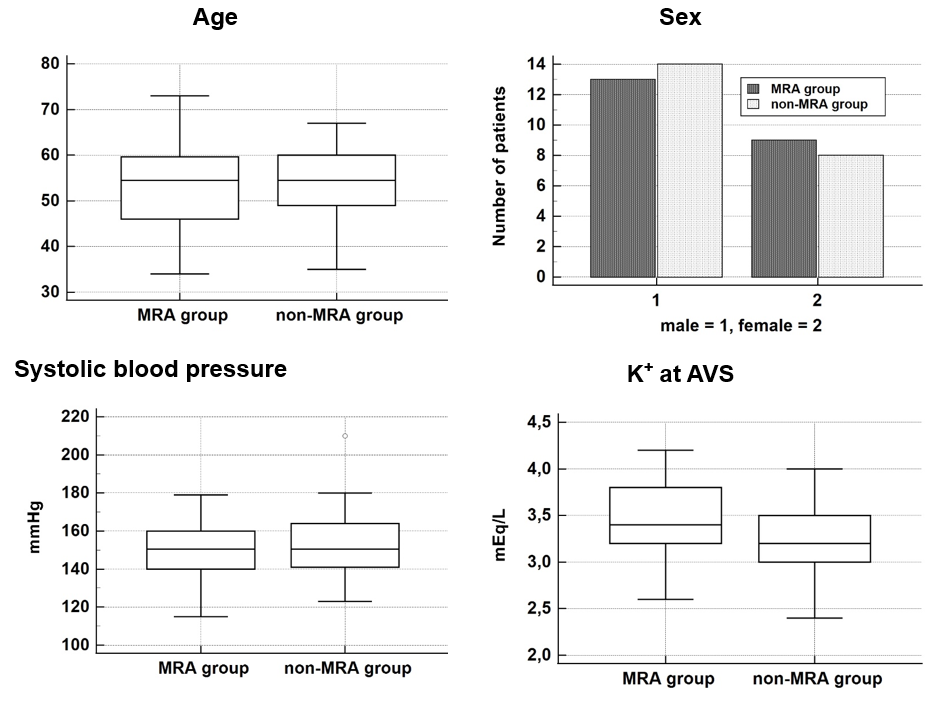


Model 4. Matching 1:3. Variables were age, sex, systolic blood pressure (SBP), serum potassium, defined daily dose (DDD), imaging positive for nodules; missing values were calculated using the mean of the series.


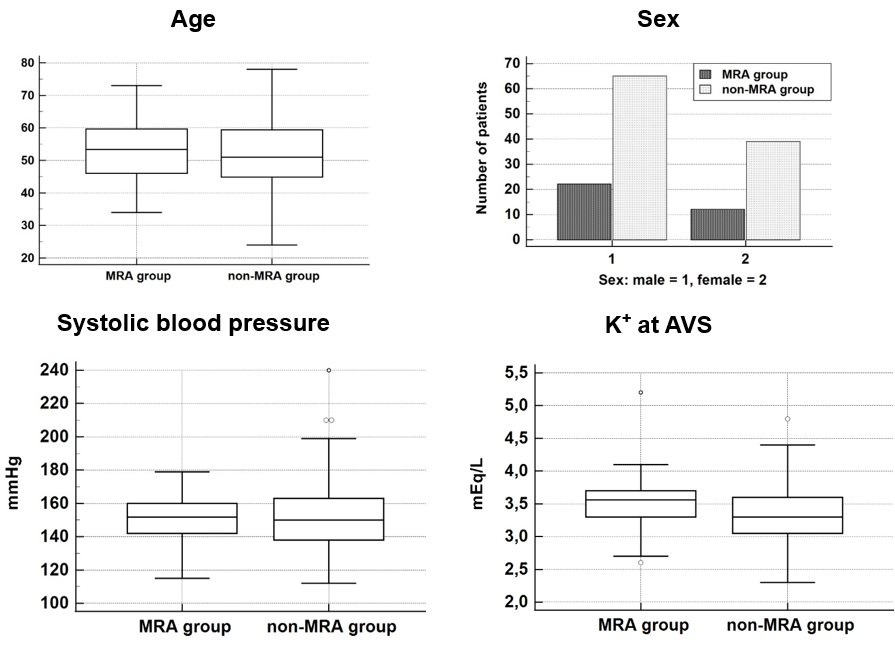


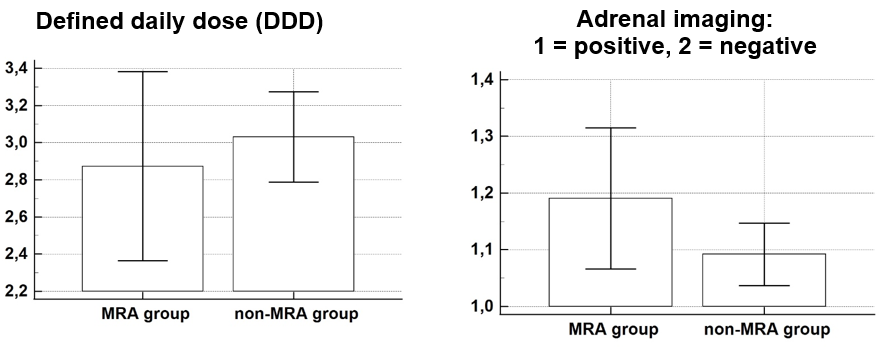


**Lateralization index (LI) and rate of lateralization by chi-square test and by conditional regression model, after propensity score matching (PSM).**

**Lateralization index (LI) after propensity score matching (PSM).**

**Model 1**

|  | **MRA**  **(n = 22)** | **P value** | **Non-MRA**  **(n = 22)** | **Total**  **(n = 44)** |
| --- | --- | --- | --- | --- |
| **Unstimulated AVS - LI** | 8.3 [2.0-26.7] | 0.25 | 3.8 [1.8-8.4] | 4.6 [1.9-20.1] |

**Model 2**

|  | **MRA**  **(n = 22)** | **P value** | **Non-MRA**  **(n = 22)** | **Total**  **(n = 44)** |
| --- | --- | --- | --- | --- |
| **Unstimulated AVS - LI** | 8.3 [2.0-26.7] | 0.26 | 4.5 [1.8-11.2] | 5.3 [1.9-17.7] |

**Model 3**

|  | **MRA**  **(n = 22)** | **P value** | **Non-MRA**  **(n = 22)** | **Total**  **(n = 44)** |
| --- | --- | --- | --- | --- |
| **Unstimulated AVS - LI** | 8.3 [2.0-26.7] | 0.54 | 6.9 [1.4-23.8] | 6.9 [1.8-23.8] |

**Model 4**

|  | **MRA**  **(n = 34)** | **P value** | **Non-MRA**  **(n = 104)** | **Total**  **(n = 138)** |
| --- | --- | --- | --- | --- |
| **Unstimulated AVS - LI** | 6.0 [2.3-26.7] | 0.5 | 11.2 [2.6-29.6] | 8.4 [2.6-27.9] |

**Rate of lateralization by chi-square test after propensity score matching (PSM).**

**Model 1**

|  | | | | | **MRA at AVS:**  1 = yes, 2 = no | | Total | |
| --- | --- | --- | --- | --- | --- | --- | --- | --- |
|  |  |  |  |  | 1 | 2 |  |  |
| **Lateralization by center's cut-off** | 1,00 | Count | | | 17 | 10 | 27 | |
|  |  | Expected Count | | | 13,5 | 13,5 | 27,0 | |
|  |  | % within lateralization yes= 1, no = 2 | | | 63,0% | 37,0% | 100,0% | |
|  |  | % within MRA at AVS | | | 77,3% | 45,5% | 61,4% | |
|  |  | % of Total | | | 38,6% | 22,7% | 61,4% | |
|  | 2,00 | Count | | | 5 | 12 | 17 | |
|  |  | Expected Count | | | 8,5 | 8,5 | 17,0 | |
|  |  | % within lateralization yes= 1, no = 2 | | | 29,4% | 70,6% | 100,0% | |
|  |  | % within MRA at AVS | | | 22,7% | 54,5% | 38,6% | |
|  |  | % of Total | | | 11,4% | 27,3% | 38,6% | |
| Total | | Count | | | 34 | 22 | 22 | |
|  |  | Expected Count | | | 34,0 | 22,0 | 22,0 | |
|  |  | % within lateralization yes= 1, no = 2 | | | 24,6% | 50,0% | 50,0% | |
|  |  | % within MRA at AVS | | | 100,0% | 100,0% | 100,0% | |
|  |  | % of Total | | | 24,6% | 50,0% | 50,0% | |
| Chi-square test | | | | | | | |  |
|  | **Value** | | **df** | **Asymptotic Significance (2-sided)** | **Exact Sig. (2-sided)** | **Exact Sig. (1-sided)** | |  |
| **Pearson Chi-Square** | 4,697 | | 1 | 0,030 |  |  | |  |
| **Continuity Correction** | 3,451 | | 1 | 0,063 |  |  | |  |
| **Likelihood Ratio** | 4,806 | | 1 | 0,028 |  |  | |  |
| **Fisher's Exact Test** |  | |  |  | 0,062 | 0,031 | |  |
| **Linear-by-Linear Association** | 4,590 | | 1 | 0,032 |  |  | |  |
| **N of Valid Cases** | 44 | |  |  |  |  | |  |

**Model 2**

|  | | | | | **MRA at AVS:**  1 = yes, 2 = no | | | | **Total** |
| --- | --- | --- | --- | --- | --- | --- | --- | --- | --- |
|  |  |  |  |  | **1** | | **2** | |  |
| **Lateralization by center's cut-off** | **1,00** | Count | | | 17 | | 12 | | 29 |
|  |  | Expected Count | | | 14,5 | | 14,5 | | 29,0 |
|  |  | % within lateralization yes= 1, no = 2 | | | 58,6% | | 41,4% | | 100,0% |
|  |  | % within MRA at AVS | | | 77,3% | | 54,5% | | 65,9% |
|  |  | % of Total | | | 38,6% | | 27,3% | | 65,9% |
|  | **2,00** | Count | | | 5 | | 10 | | 15 |
|  |  | Expected Count | | | 7,5 | | 7,5 | | 15,0 |
|  |  | % within lateralization yes= 1, no = 2 | | | 33,3% | | 66,7% | | 100,0% |
|  |  | % within MRA at AVS | | | 22,7% | | 45,5% | | 34,1% |
|  |  | % of Total | | | 11,4% | | 22,7% | | 34,1% |
| Total | | Count | | | 22 | | 22 | | 44 |
|  |  | Expected Count | | | 22,0 | | 22,0 | | 44,0 |
|  |  | % within lateralization yes= 1, no = 2 | | | 50,0% | | 50,0% | | 100,0% |
|  |  | % within MRA at AVS | | | 100,0% | | 100,0% | | 100,0% |
|  |  | % of Total | | | 50,0% | | 50,0% | | 100,0% |
| Chi square test | | | | | | | | | |
|  | **Value** | | **df** | **Asymptotic Significance (2-sided)** | | **Exact Sig. (2-sided)** | | **Exact Sig. (1-sided)** | |
| **Pearson Chi-Square** | 2,529 | | 1 | 0,112 | |  | |  | |
| **Continuity Correction** | 1,618 | | 1 | 0,203 | |  | |  | |
| **Likelihood Ratio** | 2,565 | | 1 | 0,109 | |  | |  | |
| **Fisher's Exact Test** |  | |  |  | | 0,203 | | 0,101 | |
| **Linear-by-Linear Association** | 2,471 | | 1 | 0,116 | |  | |  | |
| **N of Valid Cases** | 44 | |  |  | |  | |  | |

**Model 3**

|  | | | | | | **MRA at AVS:**  1 = yes, 2 = no | | | **Total** |
| --- | --- | --- | --- | --- | --- | --- | --- | --- | --- |
|  |  |  |  |  |  | **1** | **2** | |  |
| **Lateralization by center's cut-off** | **1,00** | | Count | | | 17 | 13 | | 30 |
|  |  |  | Expected Count | | | 14,5 | 15,0 | | 30,0 |
|  |  |  | % within lateralization yes= 1, no = 2 | | | 58,6% | 43,3% | | 100,0% |
|  |  |  | % within MRA at AVS | | | 77,3% | 59,1% | | 68,2% |
|  |  |  | % of Total | | | 38,6% | 29,5% | | 68,2% |
|  | **2,00** | | Count | | | 5 | 9 | | 14 |
|  |  |  | Expected Count | | | 7,5 | 7,0 | | 14,0 |
|  |  |  | % within lateralization yes= 1, no = 2 | | | 33,3% | 64,3% | | 100,0% |
|  |  |  | % within MRA at AVS | | | 22,7% | 40,9% | | 31,8% |
|  |  |  | % of Total | | | 11,4% | 20,5% | | 31,8% |
| Total | | | Count | | | 22 | 22 | | 44 |
|  |  |  | Expected Count | | | 22,0 | 22,0 | | 44,0 |
|  |  |  | % within lateralization yes= 1, no = 2 | | | 50,0% | 50,0% | | 100,0% |
|  |  |  | % within MRA at AVS | | | 100,0% | 100,0% | | 100,0% |
|  |  |  | % of Total | | | 50,0% | 50,0% | | 100,0% |
| Chi square test | | | | | | | | | |
|  | | **Value** | | **df** | **Asymptotic Significance (2-sided)** | | **Exact Sig. (2-sided)** | **Exact Sig. (1-sided)** | |
| **Pearson Chi-Square** | | 1,676 | | 1 | 0,195 | |  |  | |
| **Continuity Correction** | | 0,943 | | 1 | 0,332 | |  |  | |
| **Likelihood Ratio** | | 1,694 | | 1 | 0,193 | |  |  | |
| **Fisher's Exact Test** | |  | |  |  | | 0,332 | 0,166 | |
| **Linear-by-Linear Association** | | 1,638 | | 1 | 0,201 | |  |  | |
| **N of Valid Cases** | | 44 | |  |  | |  |  | |

**Model 4**

|  | | | | | **MRA at AVS:**  1 = yes, 2 = no | | Total | |
| --- | --- | --- | --- | --- | --- | --- | --- | --- |
|  |  |  |  |  | 1 | 2 |  |  |
| **Lateralization by center's cut-off** | 1,00 | Count | | | 28 | 77 | 105 | |
|  |  | Expected Count | | | 25,9 | 79,1 | 105,0 | |
|  |  | % within lateralization yes= 1, no = 2 | | | 26,7% | 73,3% | 100,0% | |
|  |  | % within MRA at AVS | | | 82,4% | 74,0% | 76,1% | |
|  |  | % of Total | | | 20,3% | 55,8% | 76,1% | |
|  | 2,00 | Count | | | 6 | 27 | 33 | |
|  |  | Expected Count | | | 8,1 | 24,9 | 33,0 | |
|  |  | % within lateralization yes= 1, no = 2 | | | 18,2% | 81,8% | 100,0% | |
|  |  | % within MRA at AVS | | | 17,6% | 26,0% | 23,9% | |
|  |  | % of Total | | | 4,3% | 19,6% | 23,9% | |
| Total | | Count | | | 34 | 104 | 138 | |
|  |  | Expected Count | | | 34,0 | 104,0 | 138,0 | |
|  |  | % within lateralization yes= 1, no = 2 | | | 24,6% | 75,4% | 100,0% | |
|  |  | % within MRA at AVS | | | 100,0% | 100,0% | 100,0% | |
|  |  | % of Total | | | 24,6% | 75,4% | 100,0% | |
| *Chi-square test* | | | | | | | |  |
|  | **Value** | | **df** | **Asymptotic Significance (2-sided)** | **Exact Sig. (2-sided)** | **Exact Sig. (1-sided)** | |  |
| **Pearson Chi-Square** | 0,974 | | 1 | 0,324 |  |  | |  |
| **Continuity Correction** | 0,570 | | 1 | 0,450 |  |  | |  |
| **Likelihood Ratio** | 1,021 | | 1 | 0,312 |  |  | |  |
| **Fisher's Exact Test** |  | |  |  | 0,365 | 0,228 | |  |
| **Linear-by-Linear Association** | 0,966 | | 1 | 0,326 |  |  | |  |
| **N of Valid Cases** | 138 | |  |  |  |  | |  |

**Rate of lateralization by conditional regression model (Omnibus Tests of Model Coefficients) after propensity score matching (PSM).** Please note that the negative value of the beta coefficient indicates a higher rate of lateralization in the MRA group, due to coding of MRA patients with lateralization at AVS =1 and non-MRA patients with lateralization at AVS = 2, as indicated on the IBM website.

**Model 1**

| -2 Log Likelihood | Overall (score) | | | | Change From Previous Step | | | | | | Change From Previous Block | | | | |  |
| --- | --- | --- | --- | --- | --- | --- | --- | --- | --- | --- | --- | --- | --- | --- | --- | --- |
|  | Chi-square | df | Sig. | | Chi-square | | df | | Sig. | | Chi-square | | df | | Sig. |  |
| 0,334 | 11,000 | 1 | <0,001 | | 14,915 | | 1 | | <0,001 | | 14,915 | | 1 | | <0,001 |  |
| Variables in the Equation | | | | | | | | | | | | | | | | |
|  | B | SE | | Wald | | df | | Sig. | | Exp(B) | | 95,0% CI for Exp(B) | | | | |
|  |  |  |  |  |  |  |  |  |  |  |  | Lower | | Upper | | |
| MRA at AVS | -4,179 | 2,474 | | 2,854 | | 1 | | 0,091 | | 0,015 | | 0,000 | | 1,953 | | |

**Model 2**

| -2 Log Likelihood | Overall (score) | | | | | Change From Previous Step | | | | | | Change From Previous Block | | | | |
| --- | --- | --- | --- | --- | --- | --- | --- | --- | --- | --- | --- | --- | --- | --- | --- | --- |
|  | Chi-square | | df | Sig. | | Chi-square | | df | | Sig. | | Chi-square | | df | | Sig. |
| 0,365 | 12,000 | | 1 | <0,001 | | 16,271 | | 1 | | <0,001 | | 16,271 | | 1 | | <0,001 |
| Variables in the Equation | | | | | | | | | | | | | | | | |
|  | | B | SE | | Wald | | df | | Sig. | | Exp(B) | | 95,0% CI for Exp(B) | | | |
|  |  |  |  |  |  |  |  |  |  |  |  |  | Lower | | Upper | |
| MRA at AVS | | -4,179 | 2,368 | | 3,113 | | 1 | | 0,078 | | 0,015 | | 0,000 | | 1,589 | |

**Model 3**

| -2 Log Likelihood | Overall (score) | | | | | Change From Previous Step | | | | | | Change From Previous Block | | | | |
| --- | --- | --- | --- | --- | --- | --- | --- | --- | --- | --- | --- | --- | --- | --- | --- | --- |
|  | Chi-square | | df | Sig. | | Chi-square | | df | | Sig. | | Chi-square | | df | | Sig. |
| 0,395 | 13,000 | | 1 | <0,001 | | 17,627 | | 1 | | <0,001 | | 17,627 | | 1 | | <0,001 |
| Variables in the Equation | | | | | | | | | | | | | | | | |
|  | | B | SE | | Wald | | df | | Sig. | | Exp(B) | | 95,0% CI for Exp(B) | | | |
|  |  |  |  |  |  |  |  |  |  |  |  |  | Lower | | Upper | |
| MRA at AVS | | -4,179 | 2,275 | | 3,373 | | 1 | | 0,066 | | 0,015 | | 0,000 | | 1,324 | |

**Model 4**

| -2 Log Likelihood | Overall (score) | | | | | Change From Previous Step | | | | | | Change From Previous Block | | | | | |
| --- | --- | --- | --- | --- | --- | --- | --- | --- | --- | --- | --- | --- | --- | --- | --- | --- | --- |
|  | Chi-square | | df | Sig. | | Chi-square | | df | | Sig. | | Chi-square | | df | | Sig. | |
| 5,788 | 8,000 | | 1 | 0,005 | | 10,847 | | 1 | | <0,001 | | 10,847 | | 1 | | <0,001 | |
| Variables in the Equation | | | | | | | | | | | | | | | | |  |
|  | | B | SE | | Wald | | df | | Sig. | | Exp(B) | | 95,0% CI for Exp(B) | | | |  |
|  |  |  |  |  |  |  |  |  |  |  |  |  | Lower | | Upper | |  |
| MRA at AVS | | -4,179 | 2,901 | | 2,076 | | 1 | | 0,150 | | 0,015 | | 0,000 | | 4,509 | |  |

**Rate of uPA identification in MRA and non-MRA patients by DRC subgroup in bilaterally selective unstimulated AVS: A) patients with undetectable renin levels (DRC ≤ 2 mIU/L), B) suppressed renin levels (DRC < 8.2 mIU/L), and C) unsuppressed renin levels (DRC ≥ 8.2 mIU/L).**

**A) patients with undetectable renin levels (DRC ≤ 2 mIU/L).**

| ***Case Processing Summary*** | | |
| --- | --- | --- |
|  |  | ***Valid N (listwise)*** |
| ***MRA group = 1*** | ***uPA = 1*** | 11 |
|  | ***non-uPA = 2*** | 1 |
| ***Non-MRA group = 2*** | ***uPA = 1*** | 89 |
|  | ***non-uPA = 2*** | 64 |
| **Valid** |  | 165 |
| **Missing** |  | 16 |
| **Total** |  | 181 |

***
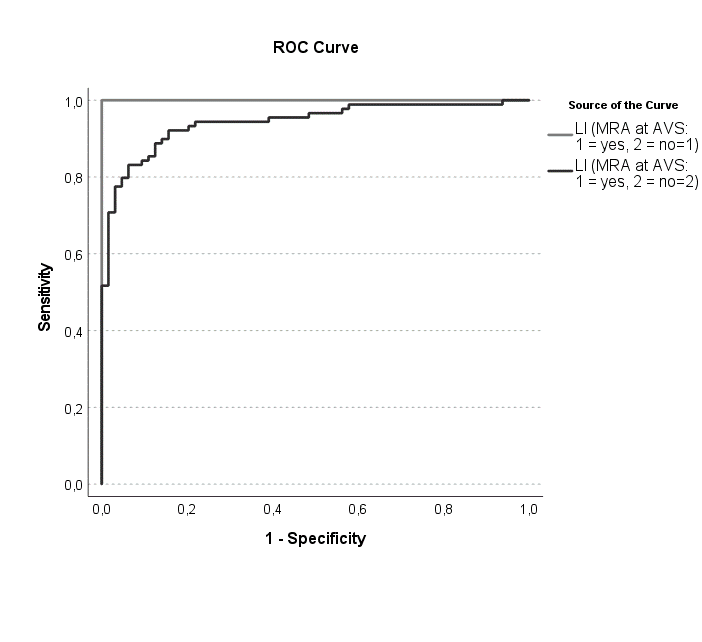
***

| ***Area Under the ROC Curve*** | |
| --- | --- |
| ***MRA at AVS*** | ***Area*** |
| MRA = 1 | 1,000 |
| Non-MRA = 2 | 0,942 |

| ***Independent-Group Area Difference Under the ROC Curve*** | | | | | |
| --- | --- | --- | --- | --- | --- |
| ***Asymptotic*** | | ***AUC Difference*** | ***Std. Error Difference*** | ***Asymptotic 95% Confidence Interval*** | |
| ***z*** | ***Sig. (2-tail)*** |  |  | ***Lower Bound*** | ***Upper Bound*** |
| 3,137 | 0,002 | 0,058 | 0,018 | 0,022 | 0,094 |

| **Unstimulated LI – DRC ≤ 2 mIU/L**  **unilateral PA vs. non-unilateral PA** | **MRA group** | **Non-MRA group** |
| --- | --- | --- |
| Number of cases (positive/negative)  Associated criterion  Youden Index  AUROC (95% CI) | 12 (11/1) | 153 (89/64)  >2.42  0.765  0.942 (0.892-0.973) |

Abbreviations: AUROC, area under receiving operating characteristic (ROC) curve; 95% CI, confidence interval; DRC, direct renin concentration; LI, Lateralization Index, MRA, mineralocorticoid receptor antagonist.

**B) patients with suppressed renin levels (DRC < 8.2 mIU/L).**


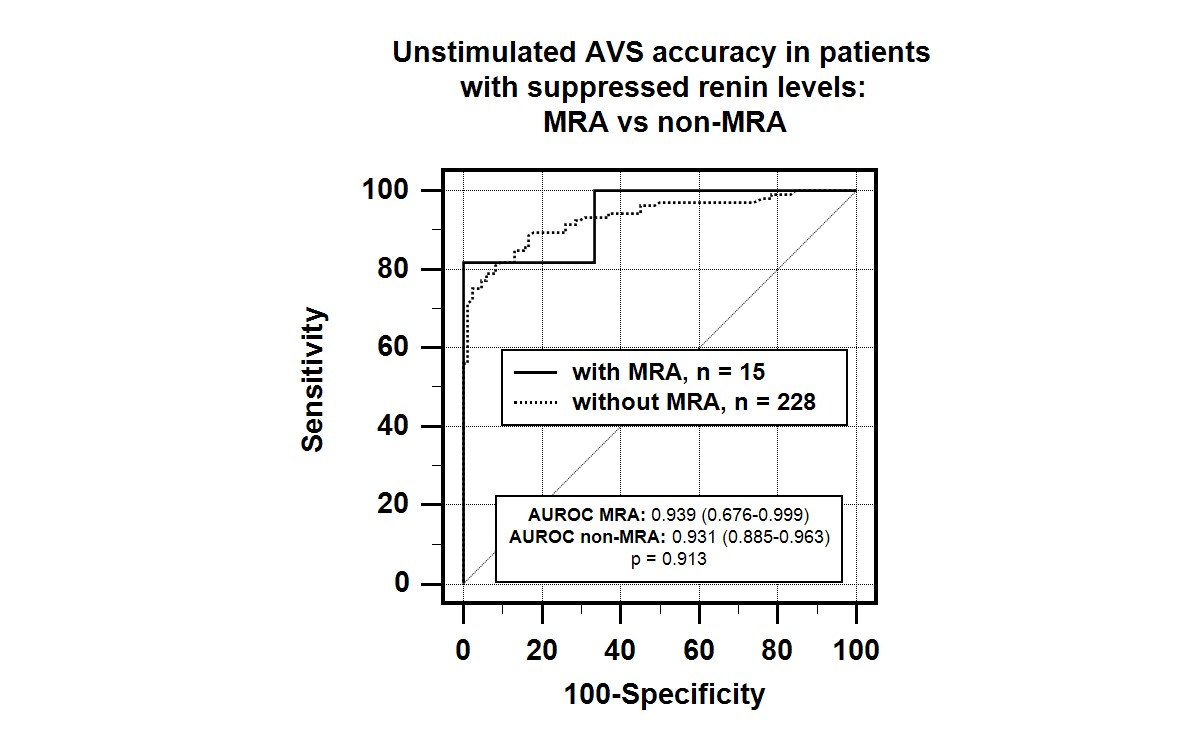


| **LI in unilateral PA vs. non-unilateral PA** | **MRA group** | **Non-MRA group** | **P value** |
| --- | --- | --- | --- |
| **Unstimulated LI – DRC ≤ 2 mIU/L**  **unilateral PA vs. non unilateral PA**  Number of cases (positive/negative)  Associated criterion  Youden Index  AUROC (95% CI) | 14 (11/3)  >3.05  0.818  0.939 (0.676-0.999) | 189 (105/84)  >3.47  0.731  0.931 (0.885-0.963) | 0.913 |

The P value is for AUROC comparison between the MRA and the non-MRA group. Abbreviations: AUROC, area under receiving operating characteristic (ROC) curve; 95% CI, confidence interval; DRC, direct renin concentration; LI, Lateralization Index, MRA, mineralocorticoid receptor antagonist; PA, primary aldosteronism.

**C) patients with unsuppressed renin levels (DRC ≥ 8.2 mIU/L).**


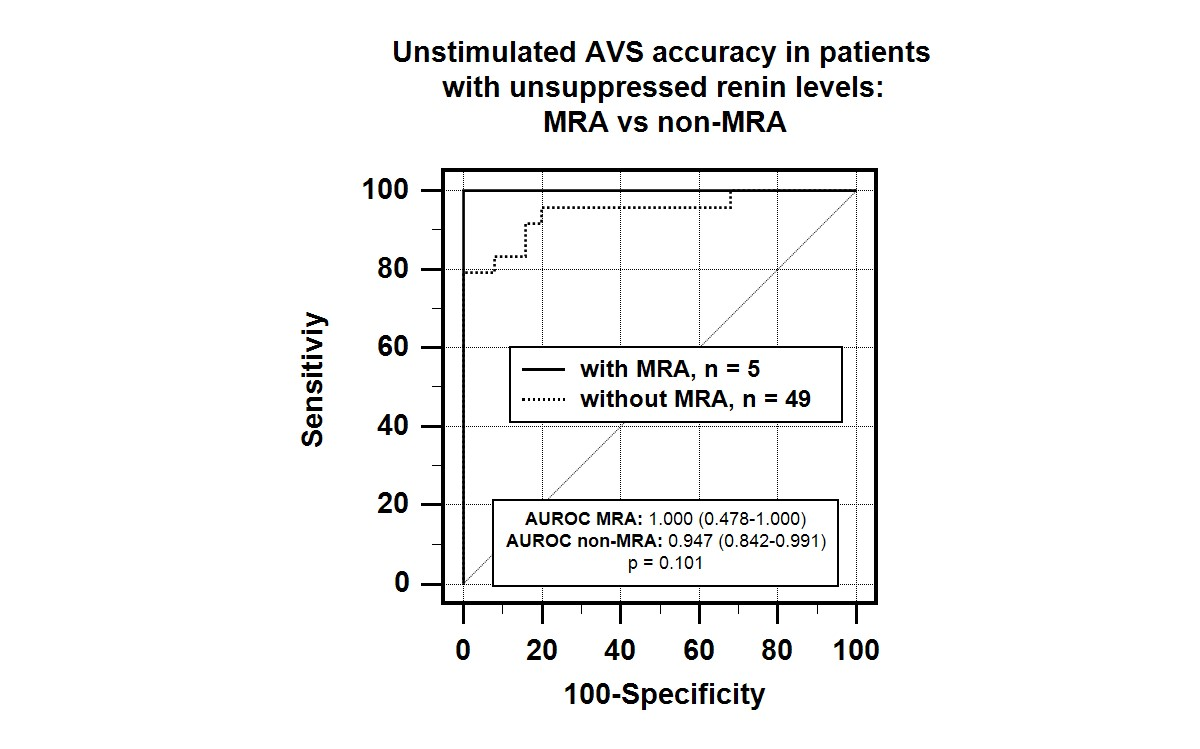


| **LI in unilateral PA vs. non-unilateral PA** | **MRA group** | **Non-MRA group** | **P value** |
| --- | --- | --- | --- |
| **Unstimulated LI – DRC ≥ 8.2 mIU/L**  **unilateral PA vs. non unilateral PA**  Number of cases (positive/negative)  Associated criterion  Youden Index  AUROC (95% CI) | 5 (3/2)  >1.34  1.000  1.000 (0.478-1.000) | 49 (24/25)  >3.35  0.792  0.947 (0.842-0.991) | 0.101 |

The P value is for AUROC comparison between the MRA and the non-MRA group. Abbreviations: AUROC, area under receiving operating characteristic (ROC) curve; 95% CI, confidence interval; DRC, direct renin concentration; LI, Lateralization Index, MRA, mineralocorticoid receptor antagonist; PA, primary aldosteronism.

**Supplemental References**

1. Bossuyt PM, Reitsma JB, Bruns DE, Gatsonis CA, Glasziou PP, Irwig L, Lijmer JG, Moher D, Rennie D, De Vet HCW, Kressel HY, Rifai N, Golub RM, Altman DG, Hooft L, Korevaar DA, Cohen JF. STARD 2015: An updated list of essential items for reporting diagnostic accuracy studies. The BMJ. 2015;351. doi:10.1136/bmj.h5527.

2. Staffa SJ, Zurakowski D. Five steps to successfully implement and evaluate propensity score matching in clinical research studies. Anesth Analg. 2018;127:1066–1073.

3. Rossi GP, Barisa M, Allolio B, Auchus RJ, Amar L, Cohen D, Degenhart C, Deinum J, Fischer E, Gordon R, Kickuth R, Kline G, Lacroix A, Magill S, Miotto D, Naruse M, Nishikawa T, Omura M, Pimenta E, Plouin PF, Quinkler M, Reincke M, Rossi E, Rump LC, Satoh F, Kool LS, Seccia TM, Stowasser M, Tanabe A, Trerotola S, Vonend O, Widimsky J, Wu KD, Wu VC, Pessina AC. The adrenal vein sampling International study (avis) for identifying the major subtypes of primary aldosteronism. Journal of Clinical Endocrinology and Metabolism. 2012;97:1606–1614.

4. Rossi GP, Rossitto G, Amar L, Azizi M, Riester A, Reincke M, Degenhart C, Widimsky J, Naruse M, Deinum J, Schultze Kool L, Kocjan T, Negro A, Rossi E, Kline G, Tanabe A, Satoh F, Christian Rump L, Vonend O, Willenberg HS, Fuller PJ, Yang J, Chee NYN, Magill SB, Shafigullina Z, Quinkler M, Oliveras A, Dun Wu K, Wu VC, Kratka Z, Barbiero G, Battistel M, Chang CC, Vanderriele PE, Pessina AC. Clinical Outcomes of 1625 Patients with Primary Aldosteronism Subtyped with Adrenal Vein Sampling. Hypertension. 2019;74:800–808

**Table S1. AVS indexes definition.**

**The Selectivity Index (SI) was defined as the ratio of the cortisol concentration in the adrenal vein of one side to that in the inferior vena cava (IVC). The Lateralization Index (LI) was defined as the ratio between the aldosterone/cortisol of the dominant side on the aldosterone/cortisol of the non-dominant side. The Relative Aldosterone Secretion Index (RASI) was defined as the adrenal vein aldosterone/cortisol ratio in the responsible side divided by the peripheral aldosterone/cortisol ratio. Successful catheterization was defined as SI ≥ each center’s cutoff, as well as presence of lateralization was defined as LI ≥ each center’s cutoff. Bilaterally selective procedures at baseline or after stimulus were defined as both left and right catheterizations having a SI ≥ each center’s cutoff.**

| **Index** | **Formula** | **Significance** |
| --- | --- | --- |
| **Selectivity Index (SI)** | 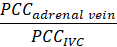 | SI estimates the correct positioning of catheters in the adrenal vein |
| **Lateralization Index (LI)** | 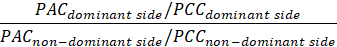 | LI measures the aldosterone secretion of the dominant over the contralateral adrenal gland corrected for the degree of selectivity and for blood dilution from extra-adrenal sources |
| **Relative Aldosterone Secretion Index (RASI)** | 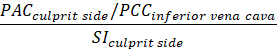 | RASI estimates the amount of aldosterone added by each adrenal to the arterial blood entering the gland corrected for the degree of AVS selectivity of that side. |

Abbreviation: PAC, plasma aldosterone concentration; PCC, plasma cortisol concentration.

**Table S2. Demographic and biochemical features of the PA patients of the Padua and the AVIS-2 cohort.** The demographic and biochemical features of the PA patients were similar in the Padua and the AVIS-2 cohort.

| **Variable** | **Padua cohort**  **(n = 121)** | **AVIS-2 cohort**  **(n = 1625)** | **P value** |
| --- | --- | --- | --- |
| **Age (years)** | 52.4±10.5 | 50.8±10.7 | 0.036 |
| **Sex (M/F), n (%)** | 74(61.2) / 47(38.8) | 995(60.6) / 640 (39.4) | 0.92 |
| **Body Mass Index (Kg/m^2^)** | 26.8±3.5 | 28.3±5.1 | 0.031 |
| **Systolic BP (mmHg)** | 148±17 | 152±20 | 0.046 |
| **Diastolic BP (mmHg)** | 89±12 | 92±13 | 0.058 |
| **Defined daily dose (DDD)** | 2.4 [1.3-3.5] | 3 [1.1-5] | 0.12 |
| **Serum K^+^ (mmol/L)** | 3.6±0.4 | 3.5±0.5 | 0.342 |
| **Potassium supplement (%)** | 82 | 54 | <0.001 |
| **DRC (mIU/L)** | 2.2 [2.0-3.9] | 2.5 [2.0-4.7] | 0.39 |
| **PAC (ng/dL)** | 18.3 [12.8-33.2] | 21.7 [13.5-34.7] | 0.36 |
| **ARR (ng/mIU)** | 68 [37-109] | 68.5 [37-120] | 0.69 |
| **Nodule dimension (mm)** | 17±14 | 15±8 | 0.09 |

Mean ± SD, or median and IQR (defined daily dose, DRC, PAC and ARR).

Abbreviations: ARR: aldosterone-to-renin-ratio; AVS: adrenal vein sampling; BP, blood pressure; DRC: direct renin concentration; PAC: plasma aldosterone concentration.

**Table S3. Rate of selective procedures per side**

| **Variable** | **Total**  **(n = 840)** | **MRA group**  **(n = 61)** | **Non-MRA group**  **(n = 779)** | **P value** |
| --- | --- | --- | --- | --- |
| **Unstimulated – right side (%)** | 83 | 86 | 83 | 0.679 |
| **Unstimulated – left side (%)** | 87 | 91 | 87 | 0.640 |
| **Post-cosyntropin – right side (%)** | 87 | 88 | 87 | 1 |
| **Post-cosyntropin – left side (%)** | 99 | 100 | 99 | 1 |

Rate of selective procedures evaluated in each side. Differences between the MRA and non-MRA groups were analyzed with Fisher exact test. Abbreviations: MRA: mineralocorticoid receptor antagonist.

**Table S4. Comparison of AUROC for LI under unstimulated conditions by active renin (DRC) levels subgroups: undetectable (≤ 2 mUI/L), suppressed (< 8.2 mUI/L) and unsuppressed (≥ 8.2 mUI/L).**

| **LI in unilateral PA vs.**  **non-unilateral PA** | **Renin Undetectable** | **Renin Suppressed** | **P value** |
| --- | --- | --- | --- |
| **Unstimulated LI – uPA vs. non-uPA**  Number of cases (positive/negative)  Associated criterion  Youden Index  AUROC (CI 95%) | 165 (100/65)  >2.55  0.757  0.942 (0.985-0.972) | 203 (116/87)  >3.47  0.736  0.932 (0.889-0.963) | 0.69 |
| **LI in unilateral PA vs.**  **non-unilateral PA** | **Renin Undetectable** | **Renin Unsuppressed** | **P value** |
| **Unstimulated LI – uPA vs. non-uPA**  Number of cases (positive/negative)  Associated criterion  Youden Index  AUROC (CI 95%) | 165 (100/65)  >2.55  0.757  0.942 (0.985-0.972) | 54 (27/27)  0.953 (0.859-0.992) | 0.73 |

Diagnostic accuracy of Lateralization Index (LI) in diagnosing unequivocally unilateral PA, versus non unilateral PA. P value referees to AUROC comparison between the renin undetectable versus suppressed and unsuppressed groups. Abbreviations: AUROC, area under receiving operating characteristic (ROC) curve; CI, confidence interval; DRC, direct renin concentration; LI, Lateralization Index; PA, primary aldosteronism.

**Table S5. Lateralization index (LI) in MRA versus non-MRA patients by active renin (DRC) level subgroups: undetectable, suppressed, and unsuppressed.**

|  | **MRA patients**  **(n = 14)** | **P value** | **Non-MRA patients**  **(n = 167)** | **Total**  **(n = 181)** |
| --- | --- | --- | --- | --- |
| **Unstimulated LI – DRC ≤ 2 mIU/L** | 10.86 [2.47-27.32] | 0.13 | 3.94 [1.73-17.13] | 4.43 [1.79-17.45] |
|  | **MRA patients**  **(n = 15)** | **P value** | **Non-MRA patients**  **(n = 213)** | **Total**  **(n = 228)** |
| **Unstimulated LI – DRC < 8.2 mIU/L** | 4.65 [2.42-13.71] | 0.53 | 3.83 [1.74-13.67] | 4.11 [1.78-13.68] |
|  | **MRA patients**  **(n = 5)** | **P value** | **Non-MRA patients**  **(n = 55)** | **Total**  **(n = 60)** |
| **Unstimulated LI – DRC ≥ 8.2 mIU/L** | 2.68 [1.22-17.18] | 0.68 | 3.8 [1.8-8.4] | 2.73 [1.56-8.23] |

The definition of LI is listed in Table S1. LI was evaluated on bilaterally selective procedures. Data are expressed as median and IQR.

Abbreviations: Direct renin concentration (DRC); Lateralization Index (LI); Mineralocorticoid Receptor Antagonists (MRA).

**Table S6. Baseline features of the 121 PA patients from the Padua cohort, subsequently divided according to the presence of MRAs at AVS. After excluding 11 patients due to other interfering therapy, a cohort of 110 PA patients could be observed.**

| **Variable** | **Total**  **(n = 110)** | **MRA group**  **(n = 30)** | **Non-MRA group**  **(n = 80)** | **P value** |
| --- | --- | --- | --- | --- |
| **Age (years)** | 52.4±10.5 | 53.4±11.0 | 51.5±10.9 | 0.478 |
| **Sex (M/F), n (%)** | 67 (60.9) / 43 (39.1) | 18 (60) / 12 (40) | 49 (61.3) / 31 (38.7) | 0.905 |
| **Body Mass Index (Kg/m^2^)** | 26.6±3.6 | 27.4±3.7 | 26.1±3.6 | 0.308 |
| **Systolic BP (mmHg)** | 148±17 | 150±17 | 147±19 | 0.553 |
| **Diastolic BP (mmHg)** | 89±12 | 93±11 | 88±13 | 0.186 |
| **Defined daily dose (DDD)** | 2.2 [1.3-3.3] | 2.0 [1.3-3.9] | 2.4 [1.3-3.0] | 0.722 |
| **Serum K^+^ (mmol/L)** | 3.6±0.4 | 3.7±0.5 | 3.5±0.4 | 0.138 |
| **Potassium supplement (%)** | 84 | 75 | 86 | 0.589 |
| **DRC (mIU/L)** | 2.2 [2.0-3.8] | 2.5 [2.0-5.5] | 2.2 [2.0-3.6] | 0.615 |
| **PAC (ng/dL)** | 18.7 [13.4-32.5] | 20.3 [14.3-36.0] | 18.3 [12.8-32.5] | 0.709 |
| **ARR (ng/mIU)** | 69.4 [36.6-113.3] | 59.3 [36.1-150.0] | 72.1 [34.2-111.7] | 0.876 |
| **Nodule dimension (mm)** | 17.0±14.3 | 15.8±8.9 | 15.6±8.9 | 0.807 |

Mean ± SD, or median and IQR (daily drug dose, DRC, PAC and ARR).

Abbreviations: ARR: aldosterone-to-renin-ratio; AVS: adrenal vein sampling; BP, blood pressure; DRC: direct renin concentration; PAC: plasma aldosterone concentration.

**Table S7. Comparison of AUROC for LI under unstimulated conditions by MRA treatment, in PA patients recruited in the Padua cohort.**

| **LI in unilateral PA vs. non unilateral PA** | **MRA group** | **Non-MRA group** | **P value** |
| --- | --- | --- | --- |
| **Unstimulated LI –**  **unilateral PA vs. non unilateral PA**  Number of cases (positive/negative)  AUROC (95% CI) | 24 (19/5)  0.947 (0.841-1.054) | 54 (45/9)  0.921 (0.849-0.993) | 0.688 |

The P value is for AUROC comparison between the MRA and the non-MRA group. Abbreviations: AUROC, area under receiving operating characteristic (ROC) curve; 95% CI, confidence interval; LI, Lateralization Index, MRA, mineralocorticoid receptor antagonist; PA, primary aldosteronism.

**Figure S1. Propensity score matching flow-chart.**


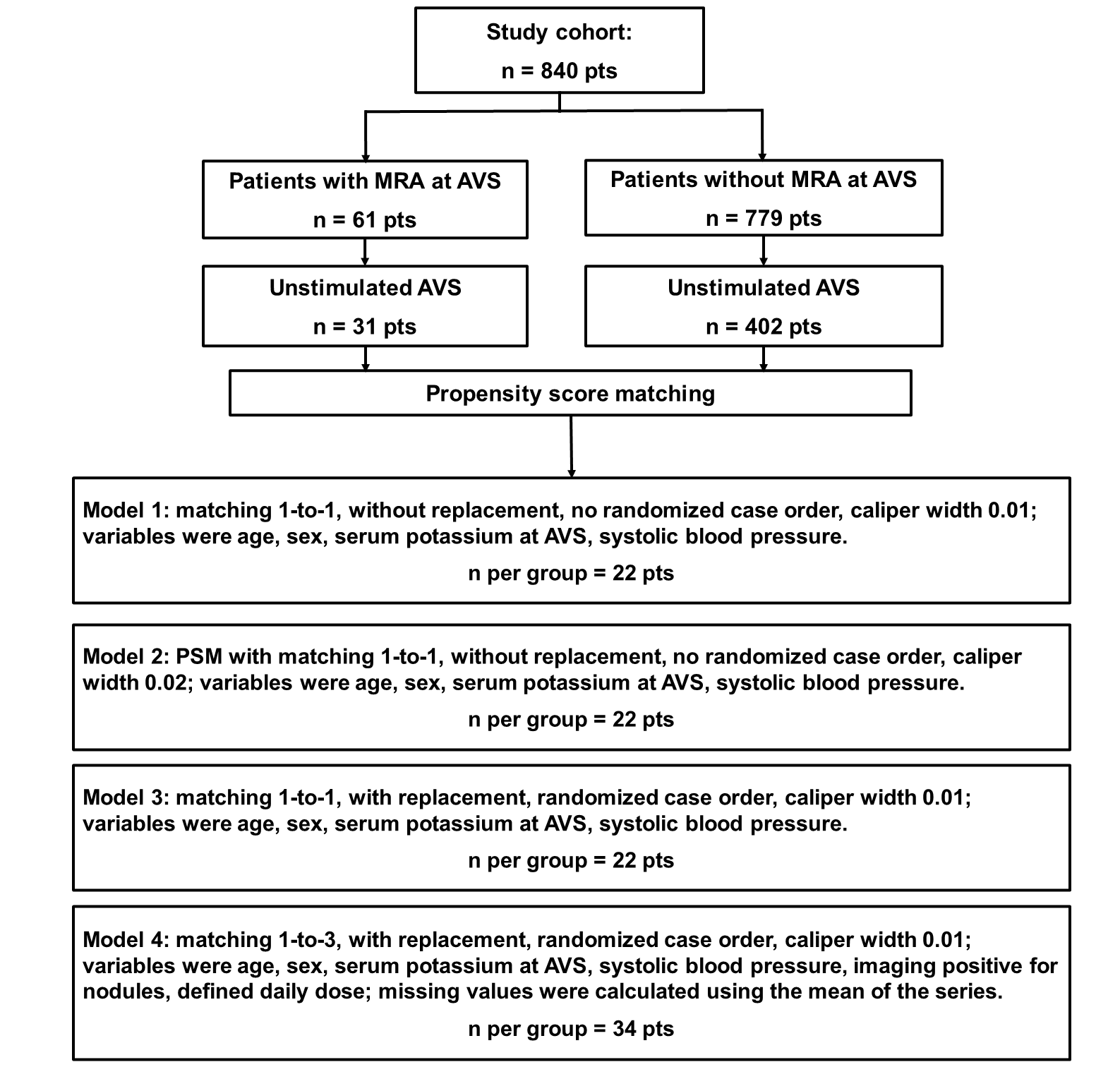

Supplement: Supplementary file 1 [file hyp-81-1391-s001.doc]
